# Concurrent Modelling and Experimental Investigation of Material Properties and Geometries Produced by Projection Microstereolithography

**DOI:** 10.3390/polym12030506

**Published:** 2020-02-26

**Authors:** Khaled G. Mostafa, Muhammad Arshad, Aman Ullah, David S. Nobes, Ahmed Jawad Qureshi

**Affiliations:** 1 Department of Mechanical Engineering, University of Alberta, Edmonton, AB T6G 1H9, Canada; kmostafa@ualberta.ca (K.G.M.); dnobes@ualberta.ca (D.S.N.); 2 Department of Agriculture Food and Nutritional Science, University of Alberta, Edmonton, AB T6G 2P5, Canada; arshad4@ualberta.ca (M.A.); ullah2@ualberta.ca (A.U.)

**Keywords:** additive manufacturing, stereolithography, working curve, accuracy, degree of curing, photopolymerization, mechanical properties, microfluidics, 3D printing, digital light projector (DLP)

## Abstract

Projection microstereolithography additive manufacturing (PµSLA-AM) systems utilize free radical photopolymerization to selectively transform liquid resins into accurate and complex, shaped, solid parts upon UV light exposure. The material properties are coupled with geometrical accuracy, implying that optimizing one response will affect the other. Material properties can be enhanced by the post-curing process, while geometry is controlled during manufacturing. This paper uses designed experiments and analytical curing models concurrently to investigate the effects of process parameters on the green material properties (after manufacturing and before applying post curing), and the geometrical accuracy of the manufactured parts. It also presents a novel accumulated energy model that considers the light absorbance of the liquid resin and solid polymer. An essential definition, named the irradiance affected zone (IAZ), is introduced to estimate the accumulated energy for each layer and to assess the feasibility of the geometries. Innovative methodologies are used to minimize the effect of irradiance irregularities on the responses and to characterize the light absorbance of liquid and cured resin. Analogous to the working curve, an empirical model is proposed to define the critical energies required to start developing the different material properties. The results of this study can be used to develop an appropriate curing scheme, to approximate an initial solution and to define constraints for projection microstereolithography geometry optimization algorithms.

## 1. Introduction

Projection microstereolithography (PµSLA) systems belong to the vat photopolymerization process, which is the “process in which liquid photopolymer in a vat is selectively cured by light-activated polymerization” according to ISO/ASTM 52900:2015(E) [1]. µSLA systems were introduced in the late 1980s as rapid prototyping equipment [2]. Currently, these systems are used for different applications like the manufacture of fully functional mechanical parts [3], microfluidics and lab on chip devices [4], as well as patient-specific medical applications [5,6,7,8]. A wide range of materials are compatible with SLA systems, such as pure polymers, mixed polymers, and ceramically loaded polymers [9,10]. The main advantage of the projection-based systems over the laser scanning system is the lower manufacturing time, as the projection systems expose the whole build area to the desired UV pattern simultaneously instead of a laser tracing each point of the pattern [11,12].

### 1.1. Principle of Operation

Like other additive manufacturing processes, the first step in PµSLA is to slice the solid model of the desired part to be manufactured. The output of this slicing process is a stack of black and white pixelated Portable Network Graphics (PNG) images and a settings file. Each image represents a cross-sectional projection for the corresponding layer. The white and black pixels represent the areas where the prepolymer resin should be polymerized or not, respectively. The number of images depends on the selected layer thickness and the part height. The minimum achievable layer thickness is determined by the minimum vertical resolution achieved by the driving system of the machine, which ranges from 1 to 100 µm in the case of PµSLA systems [8]. The input settings file contains the numerical values for the optical power (typically a UV LED), layer thickness, exposure times, and the approach and separation velocities, as well as many other parameters.

**Figure 1:**
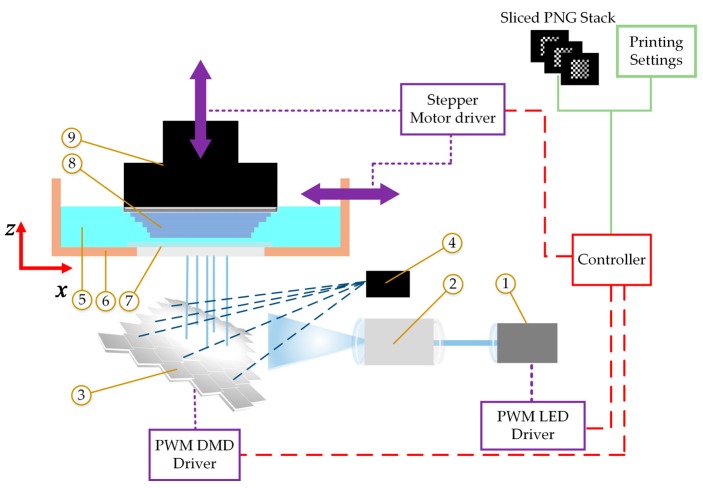
Schematic of a typical projection-based stereolithography additive manufacturing system (PµSLA).

As outlined in the schematic of a typical PµSLA system shown in Figure 1, the PNG stack and settings file are read by the machine controller, which then sends signals to the pulse width modulation (PWM) driver of the UV LED (1) to control the average LED power. The light then passes through light conditioning and expanding optics (2) for the light to be distributed equally and uniformly on the entire digital micromirrors array device (DMD) (3) [13,14]. A black pixel on the PNG image will position the corresponding micromirror to reflect the light towards a heat sink (4). A white pixel will position the corresponding micromirror to reflect the light towards the photosensitive prepolymer resin (5) in the vat (6) passing through the clear transparent PDMS window (7). The PNG image is projected via the micromirrors on the DMD to cure a complete layer of prepolymer squeezed between the previously cured layers of the part (8) and the PDMS window. The manufactured part is attached to the vertically translating build platform (9). After curing one layer, the vat moves laterally to separate the cured part from the PDMS window, then the build platform moves upwards by a distance equals to the layer thickness. PDMS inhibits free-radical polymerization by an insignificantly thin layer above its surface [11], which facilitates the separation of the part at a low separation force and minimizes part distortion [11,15]. The accuracy of the manufactured parts depends on the minimum voxel size that can be achieved. Instead of using white and black pixels, grayscale pixels can be used to control the average irradiance transmitted by each micromirror to achieve sub voxel resolution [16].

### 1.2. Photopolymerization

A successful PµSLA system, in general, requires an optimized resin formulation. The current available photosensitive resins consist of a single or a mixture of monomers and oligomers, photoinitiator, photoblocker, and enhancing additives [10,17,18,19]. The concentration of each component influences the process parameter values selected to achieve the desired material properties and geometrical accuracy [20].

When the prepolymer starts to receive the transmitted light from the DMD, the photoinitiator absorbs the light at a specific wavelength till it reaches its molar excitation threshold, then an intermolecular photocleavage occurs, and the photoinitiator decomposes to its active radicals. These radicals attack the double bonds of the surrounding monomers and oligomers, starting a chain reaction and bonding of the active monomers and oligomers with the other unsaturated ones and form polymer chains [21]. Due to this, a state of coexistence of gel and liquid appears. As more UV energy is absorbed, the gel further solidifies while retaining unconverted prepolymer trapped within the solid. The full conversion cannot be achieved during the µSLA process itself and may require post-curing [10]. An effective curing scheme achieves an acceptable amount of monomer conversion and solid phase per layer before starting a new subsequent layer to prevent shape distortion or even complete part failure.

The ideal photosensitive prepolymer resin absorbs most of the projected irradiance for each layer while allowing a small amount to penetrate to the previously manufactured layer to ensure interlayer adherence. However, in practice, a significant amount of the projected irradiance penetrates the current layer and polymerizes the uncured areas in the previous layers. The photoblocker is therefore added to minimize the irradiance penetration effect. The overall absorbance coefficient, α, at position and time (x,t) quantifies the absorbance of the light at a specific wavelength by the resin and is a function of the concentrations of the different components of the material, such that,

(1) $$\alpha \left(x,t\right)=\alpha_{I}\cdot C_{I}\left(x,t\right)+\alpha_{B}\cdot C_{B}\left(x,t\right)+\alpha_{P}\cdot \mathrm{DOC}\left(x,t\right)+\alpha_{o}\cdot \left(1-\mathrm{DOC}\left(x,t\right)\right),$$

where $\alpha_{I}$ and $\alpha_{B}$ are the light absorption coefficient of the photoinitiator and photo blocker respectively, while $C_{I}$ and $C_{B}$ are their concentrations. The photodecomposition rate of the photoinitiator or blocker, known as the photobleaching, is described as follows:

(2) $$\frac{\partial C_{j}\left(x,t\right)}{\partial t}=\beta_{j}\cdot I\left(x,t\right)\cdot C_{j,}$$

where $\beta_{j}$ is the photodecomposition coefficient of molecule j, which in this case can be either the photoinitiator or the photoblocker, and I is the irradiance. As the absorbed energy increases, the concentrations of both the photoinitiator and blocker decrease, which will increase the amount of the penetrating and non-absorbed energy [21,22]. As the degree of monomer conversion (DOC) increases, the number of prepolymer molecules decreases and the number of polymer molecules increases; usually polymer absorption, $\alpha_{P,}$ is much higher than the prepolymer molecules absorption ($\alpha_{o})$.

The Beer-Lambert equation expresses the light absorption/penetration through the material as:

(3) $$I\left(x,z\right)=I_{o}\left(x\right)\cdot e^{-\alpha \cdot z}.$$

This equation is used to derive Jacob’s working curve Equation (4) [2,23]. The I(x,z) is the irradiance at position x at depth z and $I_{o}$ is the projected irradiance at a depth equal to zero and located above the PDMS window surface and just below the prepolymer resin. The cured depth ($C_{d}),$ described by

(4) $$C_{d}\left(x\right)=D_{P}\cdot \ln\left(\frac{I\left(x\right)\cdot t}{E_{C}}\right),$$

is more practical and specially tailored for SLA as it is described in terms of material constants that can be evaluated experimentally, namely characteristic penetration depth $D_{P}$ and critical energy $E_{C}$. The critical energy is the energy at which the prepolymer start to polymerize without developing any cured depth $\left(C_{d}=0\right)$, while the characteristic penetration depth is the depth at which the exposure energy reaches $e^{-1}$ of its original value. The variables are exposure energy delivered represented by I(x)·t, where *t* is the exposure time per layer.

### 1.3. Literature Review and Problem Statement

#### 1.3.1. Material Properties

Projection microstereolithography possesses highly coupled process responses. For example, the geometrical accuracy and the material properties of the manufactured parts, which are the focus of this study, are intimately connected [23]. Manipulating the process parameters, for example the layer thickness, the exposure time, and the irradiance, to optimize one response will significantly affect all the other responses. Minimizing the effect of this coupling is one of the motivations of this study. The literature review section summarizes some of the research efforts to optimize the PµSLA process parameters to achieve different optimal response.

Aznarte et al. [24,25] studied the significance of twelve process parameters on the green mechanical properties of parts. It was found that layer thickness, exposure time, part orientation, and wait time between two successive exposures are the most significant parameters. Also, the manufactured parts showed major property anisotropy, which is confirmed by Dizon et al. [26].

Chockalingam et al. [27] studied the effect of layer thickness, orientation, and post-curing time on the strength of the part using the L18 orthogonal array and developed a second-order polynomial regression model. It was found that the layer thickness is the most significant parameter. However, the post-curing time levels chosen for this experiment were causing, rather than curing, of the polymer, which decreased its strength as the time increased.

Monzón et al. [28] studied the effect of post-curing on the anisotropy of the manufactured parts. The results showed that with proper post-curing time, the anisotropy diminishes completely along all axes with a notable increase in the mechanical strength. Also, Monzón et al. [28] showed that the position of the part on the build platform affects the mechanical properties significantly, which can be explained by the irradiance irregularities of the DMD device depicted by Zheng et al. and Warburg et al. [29,30].

Wu et al. [21] developed a curing kinetic model for acrylates-based photopolymers to predict different material properties. The test specimen was made of a single-layer part using a mould and curing light source. Yang et al. [31] developed a multi-layer curing model to estimate a theoretical average degree of curing and developed a regression model relating material properties to the degree of cure. The previous two models require extensive and expensive experimentation to evaluate all the required constants and also require detailed information about the resin components and their concentration, which is not available for most industrial resins.

#### 1.3.2. Geometrical Accuracy

Zhou et al. [32,33] used a pixel-blending optimization algorithm to improve the geometrical accuracy of the horizontal shapes. This algorithm enabled higher accuracy and sub-voxel resolution, but it did not manipulate the exposure time. Mitteramskogler et al. [34] experimentally studied the lateral growth of the dimensions with curing time.

To improve the accuracy of horizontal microchannel against the light penetration through the previously cured layers, Gong et al. [17,35] developed a multi-layer curing model to calculate the exposure time for each layer independently. O’Neill et al. [36] studied the effect of the number of layers manufactured after a microchannel on the deviation of the microchannel dimensions. These models treat the light penetration/absorption for both the liquid prepolymer and the solid polymer as the same. However they are different. As indicated by Equation (1), as the degree of monomer conversion increases, the prepolymer molecules converts to polymers, and the light absorption changes. Mostafa et al. [16] studied the effect of exposure time, grayscale, and layer thickness on the accuracy and tolerance control of cylindrical features and showed that exposure time is the most significant parameter.

Optimizing the concentrations of material components will improve the material properties and the features’ geometrical accuracy. However, excessive addition of photo-blockers to minimize light penetration through the material improves geometry but decreases strength. On the other hand, increasing photoinitiator concentration improves the material properties but decreases the critical energy for the prepolymer, which makes it highly sensitive and will result in distorted geometries [17,37,38,39]. Increasing both concentrations by insignificant amounts also increases material toxicity significantly, which makes the material unsuitable for medical applications.

### 1.4. Research Motivation and Objectives

The motivation of this study was to determine the appropriate curing scheme for producing accurate geometries with sufficient green material properties before the post-curing process, to withstand the manufacturing process and the subsequent post-processing. The geometry is mainly controlled during the manufacturing process itself, while material properties can be enhanced to an optimum with post-curing.

The coupling of the process responses entails the analysis of the effect of process parameters, namely layer thickness, exposure time, and irradiance, of both the material properties and the geometrical accuracy of the manufactured parts using a series of designed experiments. The irradiance irregularities across the building platform are identified, and their effect on the measured properties is accounted for in the experiments. A novel multi-layer curing model that differentiates between the absorbance of light through the liquid prepolymer resin and the solid polymer while calculating the accumulated energy per layer is developed and presented. A new terminology called the irradiance affected zone (IAZ) is introduced to define the number of previously cured layers affected by the exposure light of the current layer. An innovative experimental methodology for evaluating the constants of the working curve for the developed model is presented. Analogous to the working curve, this paper uses an empirical model to define the critical energy required to develop different material properties. This critical energy is a result of a logarithmic fit between the measured material properties and the numerically computed, accumulated exposure energy per layer. An experimental geometric artifact was designed to evaluate the manufacturability of different features at different sizes. Both the horizontal curing model and the vertical accumulated model are also used to assess the feasibility of manufacturing different parts.

The results obtained from this study allow a new methodology to estimate the proper curing scheme for functional parts. By knowing the different material critical energies, as defined by the empirical model, process parameters can be tuned to achieve such energies as a minimum energy constraint while also achieving required geometrical accuracy by using the different pixel-blending optimization algorithms.

## 2. Curing Analytical Models

In this section, we present the two analytical curing models we will use in the analysis of the results. The first model, which is a novel model, is called the vertical multilayer model, along the *z*-axis. This model estimates the accumulated energy received per layer. The novelty in this model is that it differentiates between the irradiance absorbance in liquid prepolymer and in solid polymer. The accumulated energy per layer is further used to model the material properties. The second model is a horizontal curing model, along the *x*-axis, which is used to study the effect of process parameters on the dimensions of different features. Both models are used to assess the manufacturing feasibility of different geometric features.

### 2.1. Vertical Multilayer Model for Accumulated Energy

**Figure 2:**
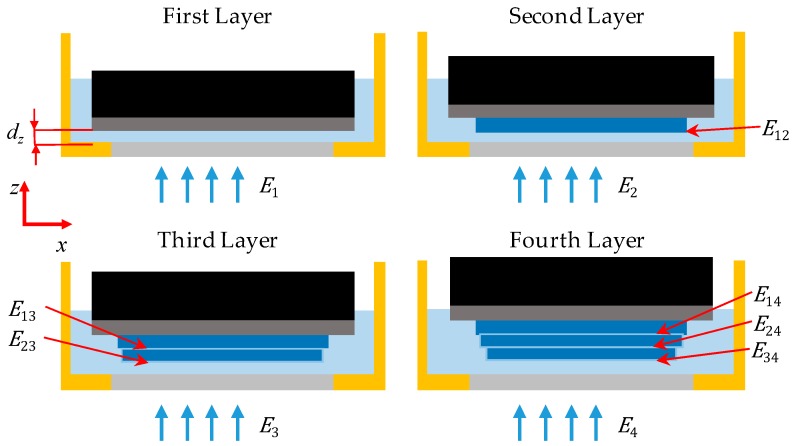
Illustration of the vertical energy accumulation model.

Various vertical energy accumulation models have been presented in different studies [35,40,41]. However, these studies assume that the irradiance absorbance is the same across liquid prepolymer and cured polymer. In reality, the absorbance coefficient differs as suggested by (1) and is shown experimentally in the next sections. The proposed new model uses two working curves to simulate the irradiance penetration through both liquid resin and cured polymer. As shown in Figure 2, the first layer of prepolymer receives its initial exposure energy, $E_{1,}$ which equals the multiplication of the irradiance I projected just above the PDMS window, for layer i, which in this case equals one, and exposure time t. During the curing of the second layer, the squeezed resin between the PDMS window and the cured layer receives the initial exposure $E_{2}$ and the first cured layer receives a portion of this exposure energy $E_{12}$ which passes through the liquid resin of layer two. Then the third layer receives $E_{3}$ and a portion of it, $E_{23,}$ penetrates through the liquid resin of the third layer and exposes the second layer, then a sub-portion of it, $E_{13},$ penetrates through the cured second layer and exposes the first layer and so on.

As described by Equation (5), each layer receives a total exposure energy of $E_{Ti}$ which is the summation of the initial exposure $E_{i}$ of the layer i, and accumulation of the penetrating exposure energy $E_{ij}$ received by layer i from the initial exposure of the subsequent layer j. The subsequent layers considered in the energy accumulation estimation are only within the irradiance affected zone (IAZ), described by Equation (7). The IAZ is the number of layers having a thickness $d_{z}$ and penetrated by irradiance I for time t before the exposure energy decreases below the critical energy $E_{c}$. For a certain layer thickness value, the IAZ is a material-dependent property and defined by the characteristic penetration depth $D_{P_{1}}$ and the critical energy $E_{c}$ of the resin. The penetrating exposure energy $E_{ij}$ from layer j to layer i, described by Equation (8), is defined as the exposure energy penetrating through one layer of liquid resin, defined by $D_{P_{1}}$, and the previously cured layers between i and j, defined by $D_{P_{2}}$. The IAZ also defines the minimum horizontal channel size, in which any horizontal gaps smaller or equal to the depth of the IAZ will cease to exist, and the vertical dimensions of the horizontal channel will deviate depending on the layer thickness used and exposure time.

(5) $$E_{Ti}=E_{i}+\overset{i+\mathrm{IAZ}}{\underset{j=i+1}{\sum }}E_{ij}$$

(6) $$E_{i}=I\cdot t$$

(7) $$IAZ=\frac{D_{P_{1}}}{d_{z}}\cdot \ln\left(\frac{I\cdot t}{E_{c}}\right)$$

(8) $$E_{ij}=E_{j}\cdot e^{-\left(\frac{d_{z}}{D_{P_{1}}}+\left(j-i-1\right)\cdot \frac{d_{z}}{D_{P_{2}}}\right)}$$

### 2.2. Horizontal Curing Model

Horizontal curing models have been presented before in several studies to optimize or evaluate horizontal geometries of a single layer [32,42,43,44]. Due to light dispersion, the projected irradiance from one micromirror on to the PDMS surface I(x) at any distance x is represented by a Gaussian profile with a Gaussian radius of $\omega_{o}$ and maximum irradiance per micromirror of $I_{m}$ as presented by Equation (9). The projector consists of a 2D array of micromirrors. However, the presented model simulates a 1D array only. The Gaussian irradiance distribution is assumed to be axisymmetric around the center of each micromirror, thus reduced into a 2D distribution. The maximum irradiance of each micromirror is assumed to be the same for the micromirrors in the model. The irradiance profile projected from a linear series of micromirrors is simulated by the superpositioning of the Gaussian profiles of all the micromirrors using (10), where $I_{T}\left(x\right)$ is the superpositioned irradiance, at any distance x, projected by N micromirrors and, P is the pitch distance between two consecutive micromirrors. This model is used to simulate the constrained surface process, where the maximum value for the cured depth cannot exceed the layer thickness $d_{z}$. The cured depth at any distance x can be estimated by using Equations (11) and (12):

(9) $$I\left(x\right)=I_{m}\cdot e^{-\frac{x^{2}}{\omega_{o}^{2}}}$$

(10) $$I_{T}\left(x\right)=\overset{N}{\underset{k=1}{\sum }}I_{m}\cdot e^{-\frac{{\left(x-k\cdot P\right)}^{2}}{\omega_{o}^{2}}}$$

(11) $$E_{T}\left(x\right)=I_{T}\left(x\right)\cdot t$$

(12) $$C_{d}\left(x\right)=\{\begin{array}{cc} D_{P_{1}}\cdot \ln\left(\frac{E_{T}\left(x\right)}{E_{C}}\right), & D_{P_{1}}\cdot \ln\left(\frac{E_{T}\left(x\right)}{E_{C}}\right)<d_{z}\\ d_{z}, & D_{P_{1}}\cdot \ln\left(\frac{E_{T}\left(x\right)}{E_{C}}\right)\geq d_{z}. \end{array}$$

**Figure 3:**
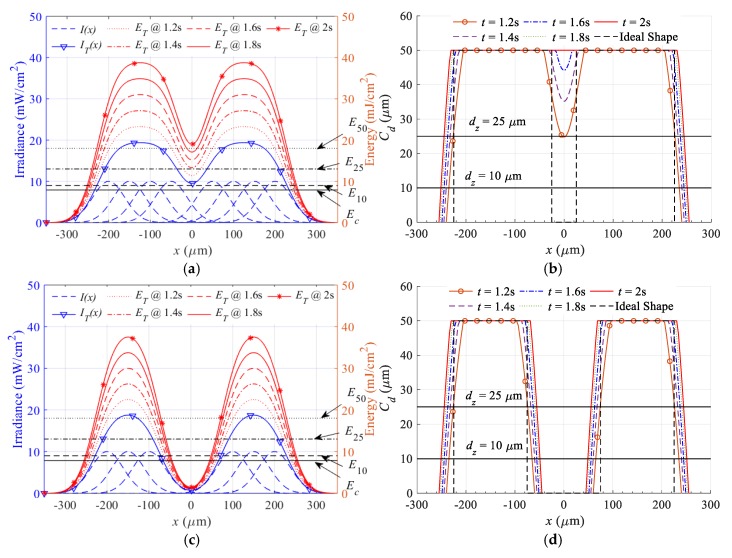
Effect of exposure time, while with one micromirror turned off, on (**a**) superposition energy (**b**) lateral dimensions, and effect of exposure time, while with three micromirrors turned off, on (**c**) superposition energy (**d**) lateral dimensions.

To simulate the effect of exposure time on the buildup exposure energy, and the lateral dimensions of simple linear features, Equations (9)–(12) are used. The outcome of this simulation is depicted in Figure 3. Figure 3a shows the results of projecting light from a linear series of nine micromirrors, with all of them turned on when the middle mirror is turned off. The dashed blue lines represent the individual Gaussian profiles reflected by each micromirror, while the solid blue line with triangles represents the superpositioned irradiance profile. The red line represents the corresponding exposure energy evaluated at different exposure times ranging from 1.2 to 2 s. The horizontal black solid lines represent the critical energy, and the other dashed black lines represent the minimum energy required to achieve a cured depth of 10, 25, and 50 µm. The cured depth for the different exposure energies is evaluated and presented in Figure 3b. The black dashed line is the ideal shape, while the black horizontal lines are representing the maximum height for the layer thickness of 10 and 25 µm. Figure 3c,d show the effect of exposure time on exposure energy and lateral dimensions for line projected by a nine micromirrors with all of them turned on when the middle three mirrors are turned off.

For the outer dimensions, as the exposure time increases, so too does the exposure energy, leading to an increase in the lateral dimensions and deviations from the ideal shape. For internal dimensions, as the exposure time increases, exposure energy also increases, leading to a decrease in the size of the internal gap until it ceases to exist, especially at lower layer thickness values. It is theoretically not possible to create an internal gap by turning off one mirror in the middle of a series of turned on mirrors. However, it is theoretically possible that an internal gap can be developed by turning off three micromirrors. This occurs when the resultant energy is above the critical energy for each layer thickness; therefore, the gap will be cured. For the three micromirrors case, the resultant energy is below the critical energy for all the layer thickness values, which means it is possible to create this gap at the studied exposure time. The superposition of irradiance in the case of linear features has lower values compared to 2D horizontal superposition, which means that a 2D gap created within a 2D feature is more challenging than within linear features. The conclusion is that there is some restriction in achieving the commercially promised horizontal resolution. An experiment is designed to evaluate the performance of these models and to study the effect of the layer thickness, exposure time, and irradiance on the dimensions of vertical and horizontal microchannels, vertical bars, and overhangs.

## 3. Materials and Methods

In order to study the concurrent influence of significant process parameters, suggested by the literature review, on the degree of monomer conversion, ultimate tensile strength, storage modulus, and geometrical accuracy, a series of characterizations are carried out based on a design of experiments. Since this work aims to relate the manufactured part characteristic to the independent process parameters, a set of experiments is carried out to determine the working curve constants of the used material and assess the irradiance of the machine at different grayscale levels and LED power.

### 3.1. Material

The prepolymer liquid resin used in this study is called PR48 clear resin, (Colorado Photopolymer Solutions, LLC, Boulder, CO, USA). This resin was chosen because it is optimized for macro and micro-scale features, and commercially available with defined chemical composition allowing development and optimization in labs. PR48 clear resin consists of Allnex Ebecryl 8210 with a 39.776 wt %, Sartomer SR 494 with a 39.776 wt % as oligomers, Esstech TPO + with 0.4 wt % as a photoinitiator, Rahn Genomer 1122 with 19.888 wt % as a reactive diluent, and Mayzo OB + with 0.16 wt % as a UV blocker [45].

### 3.2. Manufacturing Platform

The machine used in this study is the Ember^®^ DLP 3D printer (Autodesk, San Rafael, CA, USA). The LED has a maximum of 5 W and emits light at 405 nm wavelength. The DLP system has 912 × 1140 micromirrors. The build area has a maximum volume of 64 × 40 × 130 mm^3^. The machine vertical minimum resolution/layer thickness is 5 µm, and the horizontal commercial resolution is 50 µm. The machine is an open-source platform that allows complete user control over all the process parameters.

### 3.3. Irradiance Characterization

**Figure 4:**
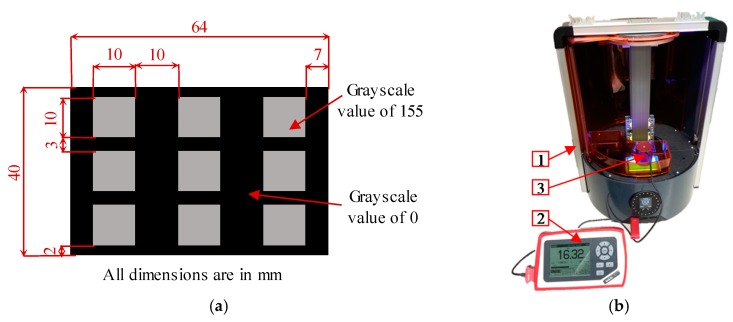
(**a**) A projected image with nine 10 × 10 mm^2^ squares having 155 grayscale pixel value, (**b**) measuring the irradiance of the Ember machine using a power meter.

Two sets of experiments are performed to characterize the irradiance of the machine. The first experiment aims to measure the irradiance, projected on the PDMS top surface, corresponding to three different LED power values at different input image grayscale values. The result of this set is used to correlate the values of the LED power and grays scale value to the irradiance value. The results will also be used in the working curve evaluation. These measurements were done by projecting a sequence of images containing nine mono-colour grayscale squares of 10 × 10 mm^2^, where the colour of the images ranges from 0 (Black) to 255 (White), as shown in Figure 4a. Then the irradiance of the Ember printer (1) was measured by a PM100 power meter (Thorlabs, Newton, NJ, USA) (2) with a photodiode power sensor Thorlabs S121C (3), as shown in Figure 4b. These measurements were repeated at three different LED power values corresponding to the pulse width modulation integer values of 255 for the maximum available power, 225, and 215; zero means the LED is off.

The second experiment is to evaluate the irradiance map irregularities across the building area in order to choose a suitable manufacturing region on the build platform with consistent irradiance for the manufacturing of the different parts. By using the same image shown in Figure 4a while projecting it at the maximum LED power and white (255) squares and measure the irradiance of each area.

### 3.4. Working Curve

**Figure 5:**
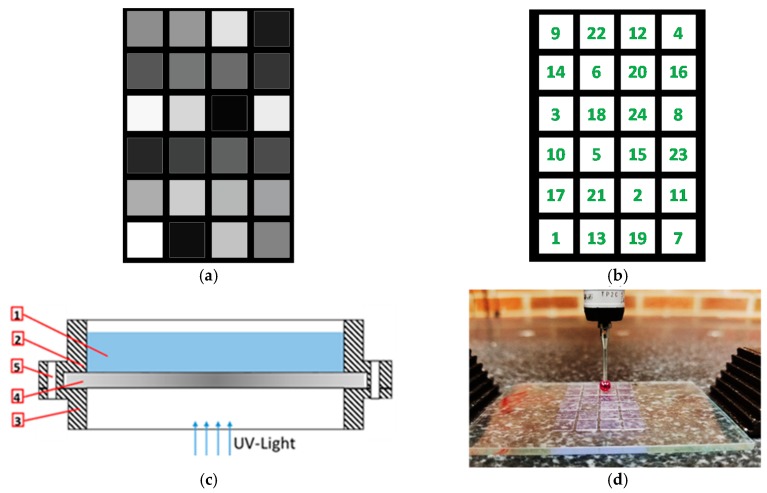
(**a**) Experiment 1: grayscale tiles for the continuous exposure-based working curve, (**b**) experiment 2: white tiles for the sequential discrete exposure-based working curve, (**c**) illustration of the special designed vat, (**d**) CMM probe measuring the cured depth of tiles height.

In order to study the effect of exposure energy on the curing depth and light penetration through the polymer, two experiments were carried out. For each of the experiments, characteristic penetration depth and critical energy are determined. The first experiment is used to measure the cured depth of the polymer formed after continuous light exposure for six seconds at different irradiance levels. This experiment is achieved by projecting an image consisting of 24 different gray-scaled tiles continuously for six seconds, as shown in Figure 5a. The lowest irradiance corresponds to a dark grey with an integer value of 11, while the highest irradiance corresponds to the white tile with an integer value of 255. This method will evaluate the light penetration and cured depth evolution through the liquid at the initial exposure of the layer.

The second experiment, which was inspired by the technique used in [46], will evaluate the cured depth and light penetration through the cured polymer. It is achieved by projecting a sequenced stack of the 24 images containing white tiles (255) only, as shown in Figure 5b. The number on each tile represents the number of images out of the 24 images that would project the specific white tile. For example, the first image will contain all the 24 tiles whereas, the second image will contain all the tiles except tile number 1, the third image will contain all the tiles except 1 and 2, and similarly, the last image will only contain tile number 24. The exposure time for each image is 200 m, and the wait time between every two successive exposures equals the typical wait time during the normal process, which is set to its optimal value of 1 s [25].

Both sets are carried out at two different LED powers: 255 (HI) and at 215 (LO). Each tile is 5 × 5 mm^2^, and the distance between any two tiles is 1 mm. The total area occupied by the 24 tiles is 23 × 35 mm^2^. The positioning of tiles in both sets is randomly assigned. The area where the tiles are distributed is limited to around 1/3 of the total build area so that the tiles are placed within an area of a tolerable irradiance map difference. A specially designed vat was used in this experiment, as shown in Figure 5c, to carry the liquid resin (1). The vat consists of the upper body (2) and lower body (3) enclosing a quartz plate (4), and the two bodies are tightened together with bolt and nut through aligned through hole (5). The upper and lower bodies were 3D printed. The unique design allows us to remove the quartz plate after each experiment, to clean the uncured resin using isopropyl alcohol spray and also to facilitate the measurements of the cured depth. The average cured depth based on three measurements on different locations per cured polymer tile was evaluated. The machine used for measurement is a Crysta-Plus M443 CMM machine (Mitutoyo, Aurora, IL, USA), which has a 0.5 µm resolution and repeatability of 4 µm. The measurement setup is shown in Figure 5d.

### 3.5. Design of Experiments for Materiall and Geometric Characterization

Three experiments were designed using a $2^{2}$ full factorial array, as shown in Table 1. Three different layer thicknesses are studied with a value of 10, 25, and 50 µm. For each experiment, the exposure time and LED power are the only variables while the layer thickness value is kept constant.

The reason for doing three separate experiments is that as the exposure time increases at lower layer thickness, the printed part adheres to the PDMS window, which halts the process in the middle and produces distorted shapes. The exposure time values were adjusted in each experiment to ensure that the maximum limit will not cause PDMS separation problems, and its lower limit will not cause part failure due to layer separation caused by incomplete curing. The LED power values used are the maximum available at 255, and 215; prolonged exposure times are required below these numbers. For each of the three experiments, the responses measured are the degree of monomer conversion, tensile strength, storage modulus, and the dimensions of several geometric features.

### 3.6. Degree of Monomer Conversion

**Figure 6:**
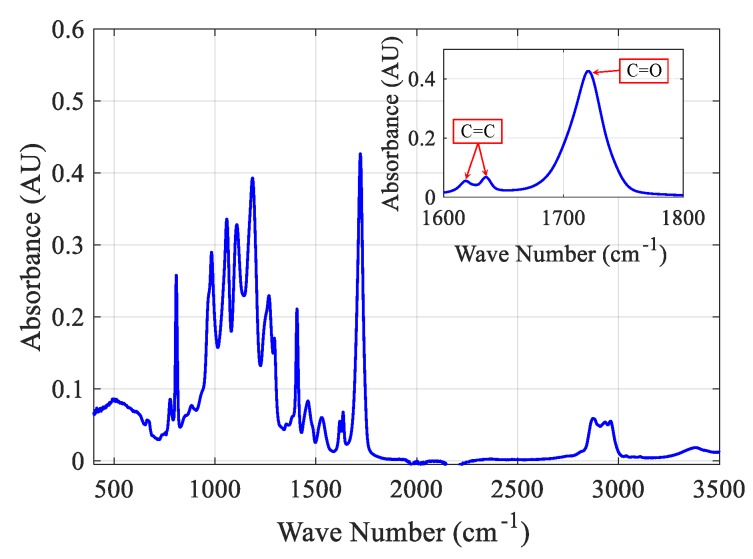
FTIR peaks considered in calculating the degree of monomer conversion.

The degree of monomer conversion (DOC) is calculated using Fourier transform infrared spectroscopy (FTIR) with attenuated total reflection (ATR) to scan both the cured polymer samples for all the experiments and the uncured prepolymer resin. Six cubes of 5 mm each were manufactured for each configuration and then appropriately cleaned with isopropyl alcohol. The cubes were then finely ground before scanning. We used a Nicolet iS50 (ThermoFisher Scientific, Waltham, MA, USA) with a build-in ATR module. Each sample was scanned 32 times with a wavenumber resolution of 2 cm^−1^. During polymerization, the double bond C=C is opened and converted to a single bond in the polymer chain. The degree of conversion can be estimated by comparing the absorbance spectra of the C=C stretching vibration peaks at 1620 cm^−1^ and 1635 cm^−1^ in the cured polymer to the same peaks in the liquid resin, as shown in Figure 6. 

The measured values are normalized against a non-variable standard bond during the reaction to account for the differences in the amount of the scanned samples. The C=O bond is chosen as the non-variable reference based on the material we have. The C=O has a stretching vibration at 1725 cm^−1^. The DOC is calculated using Equation (13):

(13) DOC = 1 − (A@1635 + A@1620)A@1725(Ao@1635 + Ao@1620)Ao@1725,

where A is the peak absorbance area of the cured sample at a specific wavenumber and $A_{o}$ is the peak absorbance area for the uncured prepolymer resin at the same specific wavenumber.

### 3.7. Mechanical Tensile Test

The ultimate tensile strength (UTS) was determined experimentally by manufacturing a standard dog bone specimen 1 BB, according to ISO 527-1:2012(E) [47,48]. This specimen was explicitly chosen due to its short overall length, which is around 30 mm and also its tight width of 4 mm with a narrow cross-section of 2 × 2 mm^2^, which makes it the smallest specimen compared to the other specimens in the ISO or ASTM. Three replicates were manufactured for each experimental configuration. The dog bones were positioned in a defined location on the build area to minimize the irradiance irregularities effects, which will allow more consistency in the results. The printing location is defined based on the irradiance measurements, which are discussed in Section 4.1. The specimens were orientated flat on the build platform, and the specimen edges were parallel to the micromirror edges. The specimens were conditioned for seven days at room temperature of 22 °C and room humidity of 23% [49]. The machine used is the 3360 series universal testing system (Instron, Norwood, MA, USA) The specimens were tensioned at an elongation rate of 0.125 mm/min.

### 3.8. Dynamic Mechanical Analysis (DMA)

A dynamic mechanical analysis (DMA) with a three-point bending test is used to measure the storage modulus corresponding to each experimental configuration. The machine used in this test is DMA Q50 (TA Instruments, New Castle, DE, USA)). The test was performed at a temperature range from 0 to 100 °C at 1 Hz cycle. The specimens are 35 × 12.5 × 4 mm^3^. In order to minimize the effect of irradiance irregularities, the specimens were manufactured within the same location that was used for the tensile test specimens and the tests were conducted in duplicate.

### 3.9. Geometrical Feature Measurement

**Figure 7:**
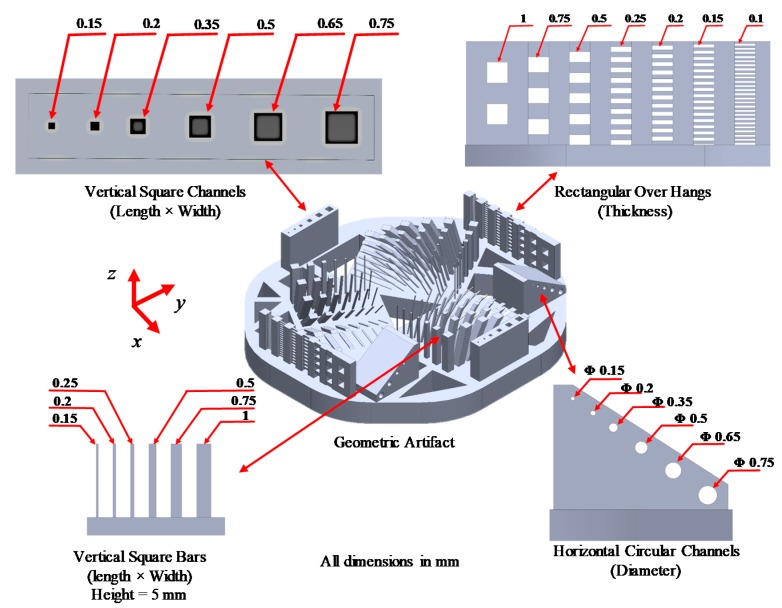
Geometric artifact to determine the minimum feasible feature size manufactured.

A geometrical artifact was designed to study the effect of process parameters on the size of different geometrical features. The artifact is designed to include four different features at different sizes to determine the minimum feasible sizes for each feature and assess the accuracy of the manufacturing process [50]. The four features included are the horizontal circular channels (the channel axis is parallel to the *x*-*z* plan), vertical square channels (the channel axis is parallel to the *z*-axis), overhangs, and vertical square bar, as shown in Figure 7. Six sizes of horizontal and vertical channels, ranging between 150 to 750 µm, are included in the artifact. While seven sizes for the square vertical bars and overhangs ranging from 100 to 1000 µm. For each size per feature, there are two replicates. The input images to the machine consist of black and white pixels only with no grayscale pixels in order to study the effect of the process parameters selected without interference from any grayscale pixels. The artifact was positioned on the platform within a defined location with tolerable irradiance irregularities. The base of the artifact was oriented flat above the build area, and the edges of the features were aligned with the micromirror edges to avoid geometrical distortion caused by the diamond orientation of the micromirrors array. The features were measured using a Stemi-508 optical microscope (Carl Zeiss, Oberkochen, Baden-Württemberg, Germany) equipped with ZEISS Axiocam 105 colour camera with 2560 × 1920 pixels, which provides a resolution of 2.5 µm at the selected optical zooming level.

## 4. Results and Discussion

### 4.1. Irradiance Characterization

**Figure 8:**
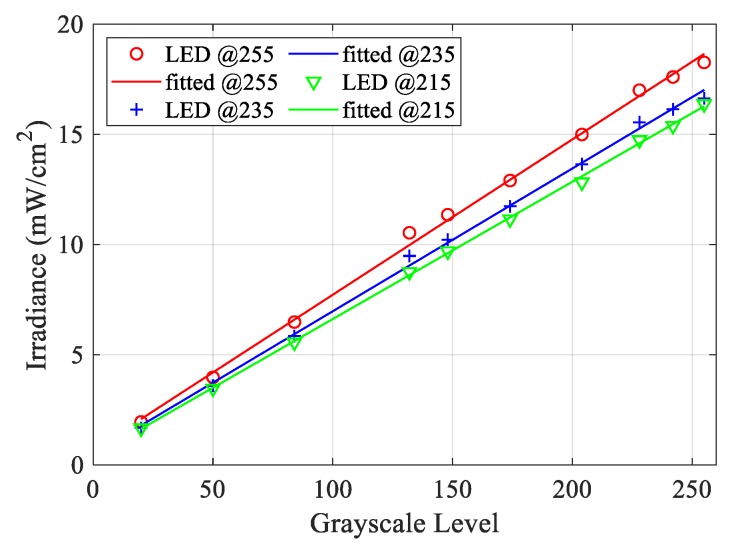
Relation between the grayscale level of the pixels and irradiance level.

The irradiance across the build area was significantly variable, as shown in Table 2. The maximum difference between the highest and the lowest regions is 6.5 mW/cm^2^. The region with the most consistent irradiance is the center column with an average of 18.2 mW/cm^2^ and was selected to manufacture the specimens of the different experiments. The value of the average irradiance of this region is used to measure the irradiance value corresponding to the different pixel grayscale colours and the LED power value. The relationship between the grayscale level of the input image’s pixels and the irradiance value is shown in Figure 8. The relation between the grayscale and the irradiance is linear. As the grayscale integer value increases, the irradiance increases. As the LED power increases, the irradiance value increases but not in a proportional trend.

### 4.2. Working Curves

**Figure 9:**
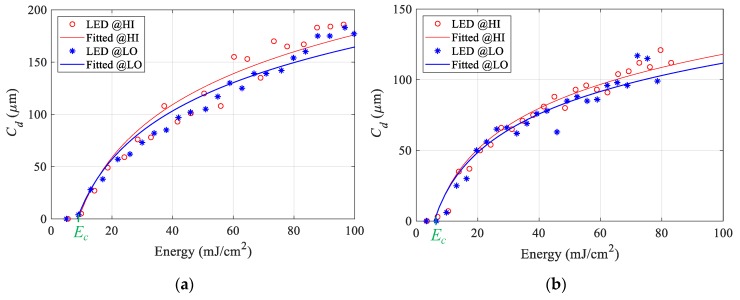
Working Curve from (**a**) continuous exposure pattern (**b**) discrete sequential pattern.

The results for the continuous light exposure pattern are depicted in Figure 9a. The critical energy $\left(E_{c}\right)$ required to initiate the photopolymerization $\left(C_{d}=0\right)$ equals 9.5 mJ/cm^2^ for both the HI and LO LED powers. On the other hand, the characteristic penetration depth $\left(D_{P}\right)$, which is the slope of this curve, if plotted on a semi-log plot, is different for the different LED power. The $D_{P}$ of the continuous pattern at high LED power is 71 µm, and at the lower power is 62 µm. Figure 9b depicts the results for the sequential discrete pattern. The critical energy $\left(E_{c}\right)$ for this pattern equals 6.5 mJ/cm^2^ for both the HI and LO LED powers. The $D_{P}$ of the discrete pattern at high LED power is 43 µm and for the lower power is 41 µm. The continuous exposure generally shows a higher $C_{d}$ for the same amount of received energy compared to the discrete pattern. The average critical energy of the two curves equal 8 mJ/cm^2^, and this energy is used in the curing models to simulate the different scenarios. The logarithmic fitted curves have an average $R^{2}$ of 0.93. The deviation between the fitted lines at the two LED power within the same the exposure pattern starts to significantly increase after 20 mJ/cm^2^ to reach approximately 15 µm at 100 mJ/cm^2^ for the continuous pattern and 5 µm at 100 mJ/cm^2^ for the sequential discrete pattern. These results show that the cured depth is sensitive to the irradiance level. The cured depth of the polymer resulting from the initial exposure is different from the cured depth formed by subsequent exposures through cured layers, for the same amount of received energy. For example, at 60 mJ/cm^2^, the difference between the cured depth for continuous exposure and the one for the sequential discrete exposure is around 50 µm. These results show that there is a difference between the absorbance of the prepolymer and the cured polymer.

### 4.3. Material Properties Characterization

**Figure 10:**
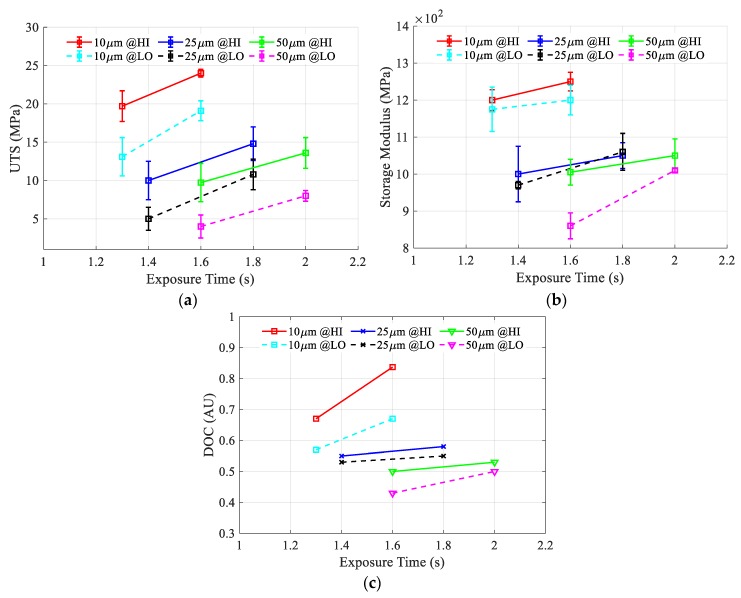
The effect of exposure time, layer thickness, and LED power on (**a**) Ultimate tensile strength, (**b**) Storage modulus, and (**c**) Degree of monomer conversion.

Figure 10a–c depicts the effect of exposure time and the ultimate tensile strength (UTS), storage modulus, and degree of monomer conversion (DOC), respectively. Each plotted line within each figure represents a particular layer thickness and LED power. There is a general trend that can be easily determined in the three figures: as the layer thickness decreases, the material properties increase, and within the same layer thickness, as the exposure time or the LED power increase, the material properties increase. For all of the properties measured, the layer thickness was found to be the most significant parameter followed by exposure time and LED power. For ultimate tensile strength, the maximum green strength achieved is 24 MPa at 10 µm layer thickness and 1.6 s exposure time at HI power, while the lowest achieved UTS was 4 MPa at 50 µm layer thickness and 1.6 s at a LO LED power. The average error is around 1.7 MPa, with a standard deviation of 0.68 MPa. The maximum achieved storage modulus is 1250 MPa at 10 µm layer thickness and 1.6 s exposure time at HI power, while the lowest achieved storage modulus was 860 MPa at 50 µm layer thickness and 1.6 s at a low LED power. The average error is around 37 MPa with a standard deviation of 19.5 MPa. For the degree of monomer conversion, the maximum achieved degree is 0.84 at 10 µm layer thickness and 1.6 s exposure time at HI power, while the lowest achieved UTS was 0.43 a 50 µm layer thickness and 1.6 s at a low LED power.

**Figure 11:**
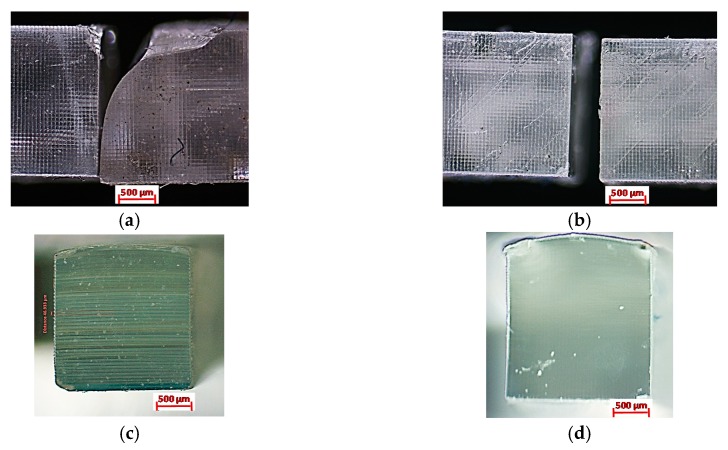
Brittle failure of the UTS specimen from the top view for (**a**) 50 µm and (**b**) 10 µm layer thickness, from the section view for (**c**) 50 µm and (**d**) 10 µm layer thickness.

During the tensile testing, the material showed a consistent brittle failure for all the tested specimens and their replicates, as shown in Figure 11a,b. The breakage is perfectly straight, with no signs of necking. The breakage happens to align with the edges of the micromirrors square array footprint on the specimen. The layer lines are visible in the cross-section of the specimen manufactured at the 50 µm-layer thickness, as shown in Figure 11c. However, the cross-section of a specimen manufactured at a 10 µm- layer thickness, as shown in Figure 11d, is almost seamless with no layers lines.

**Figure 12:**
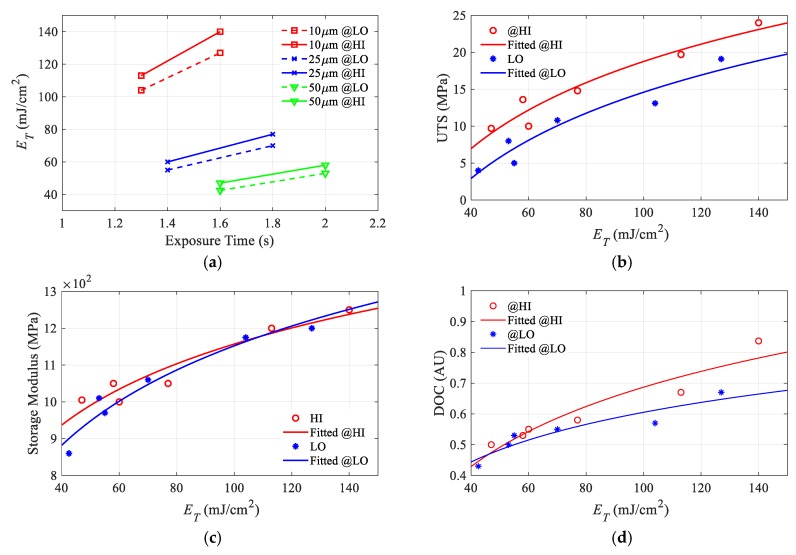
(**a**) Effect of exposure time and layer thickness on accumulated energy, Relation between accumulated energy per layer $E_{T}$ and (**b**) Ultimate tensile strength, (**c**) Storage modulus, (**d**) DOC.

The accumulated energy per layer $E_{T}$, calculated using the vertical energy accumulation model (5), is plotted against exposure time, layer thickness, and LED power, as shown in Figure 12a. This plot shows the same trend as the material properties trend with the same process parameters, as shown in Figure 10, which indicates a significant relationship between them. The maximum calculated accumulated energy per layer is 140 mJ/cm^2^ and is achieved at a layer thickness of 10 µm and a 1.6 s exposure time at HI power while the lowest is 42 mJ/cm^2^ is obtained at a layer thickness of 50 µm and a 1.6 s exposure at a low LED power.

By plotting the experimental results for any material property against the corresponding calculated accumulated energy per layer, two distinctive visible point sets are found where one of the point sets belong to the HI irradiance while the other set belongs to LO irradiance. These results suggest that for each irradiance level, there is a different trend line. Therefore, two different regression models are estimated for each LED power level per material property. For the UTS and DOC, this separation is easily determined. However, for the storage modulus, it seems that one regression model for both power level is enough, as shown in Figure 12.

We suggest an empirical regression model based on a logarithmic fitting resembling the working curve model:

(14) $$\gamma =C_{1}\cdot \ln\left(\frac{E_{T}}{C_{2}}\right),$$

where γ is a general output variable for any material property and $E_{T}$ is the calculated accumulated energy per layer (5), while $C_{1}$ and $C_{2}$ are the regression model constants. By analogy with the working curve Equation (4), $C_{1}$ will be the characteristic material property constant while $C_{2}$ is the critical energy for this property to start evolving. Using MATLAB least-squares fit function for nonlinear curves, the different constants for each material property were obtained and presented in Table 3. The average *R^2^* for all the fitted models is around 0.9. The newly defined critical energies can be used towards defining optimization constraints better than the cured depth critical energy, which does not guarantee that the part will withstand the separation forces or even its weight during printing. By knowing the projected irradiance value, the exposure time corresponding to the critical energy can be calculated and should be treated as the minimum bound for exposure time value.

### 4.4. Geometrical Features Characterization

**Figure 13:**
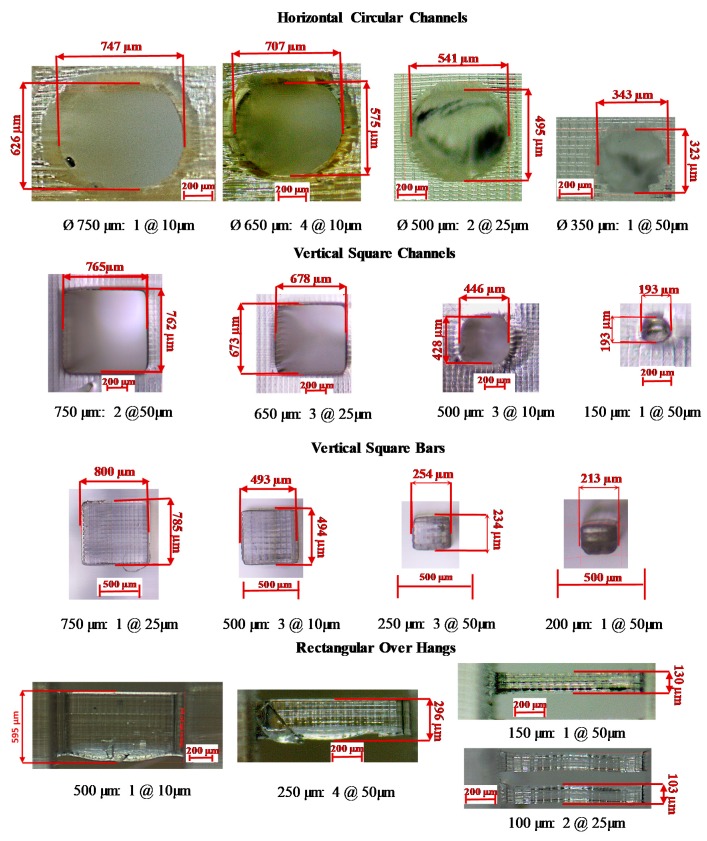
Selected optical microscope measurement images for different sizes of horizontal circular channels, vertical square channels, vertical square bars, and rectangular overhangs.

The process parameters affect the size and the form of the different features in the artifact. Figure 13 shows some optical microscope images for different sizes of horizontal circular channels, vertical square channels, vertical square bars, and rectangular overhangs, manufactured at different curing schemes. Table 4 and Table 5 present all the measured dimensions for all the manufactured features per all experimental configurations. The features that failed to be manufactured are denoted by the symbol (**×**).

For horizontal channels, the horizontal and vertical diameters were measured, with a noticeable oblong aspect ratio distortion in the manufactured geometry along the horizontal plane, as shown in Table 4. As the exposure time independently increases, the horizontal diameter of the channels decreases, as previously predicted by the horizontal curing model. Moreover, as the exposure time increases and the layer thickness decreases, the vertical diameter decreases. The minimum horizontal manufacturable channel was 350 µm diameter, achievable only by using the 50 µm layer thickness configuration. The channels with 200 and 150 µm diameters were not manufactured for any of the configurations. It was found that the channel with the 500 µm diameter was not achieved within the 10 µm layer thickness configuration. Theoretically, the 150 and the 200 µm channels are predicted to be manufacturable from the view of the horizontal curing model. However, from the accumulated energy vertical model, these channels are entirely within the IAZ, which makes them impracticable. In the configurations with a layer thickness of 50 µm, the staircase effect is visible, as shown in Figure 13. For the vertical bars, the minimum bar achieved was 100 µm, but it was not stiff enough to stand vertically. The bar was flexible enough to be bent without breaking. As the exposure time decreases, the dimension of the vertical bar also decreases, which was predicted by the horizontal curing model.

The vertical square channels are more accurate and manufacturable compared to the horizontal channels. The minimum created channel was 150 µm diameter and it was achieved within the 25 and 50 µm layer thickness configurations but not within 10 µm layer thickness ones, as shown in Table 5. It was found that, as the exposure time decreases, the dimensions of the vertical channels increase. The vertical channel dimensions depend mainly on the horizontal curing model as there is no projected irradiance from any layer within the channel gap, which means that the layer thickness does not have a significant effect compared to the exposure time. Also, as the size of the channel decreases, its shape tends to be circular rather than square, as designed. For the overhang structures, the minimum size achieved was 100 µm for both the 25 and 50 µm layer thickness configurations but not for the 10 µm layer thickness, as shown in Table 4. It was found that as the exposure time increases and the layer thickness decreases, the overhang thickness increases. An interesting phenomenon is shown in Figure 13 under the overhang section for configuration number 4 at 50 µm layer thickness. While manufacturing the 250 µm over-hang, the first layer was weak and tore during the separation. The reason for this phenomenon is that configuration 4 has the lowest exposure time and LED power for all experiments. Layer thickness and exposure time are the most significant parameters for both the horizontal channels and overhangs, while exposure time is most significant for vertical channels and bars. The LED power was the least significant parameter for all the features.

## 5. Conclusions

This paper provides a comprehensive analytical and experimental investigation of the projection microstereolithography additive manufacturing process. It studies the effect of the process parameters, namely, layer thickness, exposure time, and LED power on the ultimate tensile strength, storage modulus, degree of monomer conversion, and different micro geometrical features. A novel multilayer vertical energy accumulation model is presented, which considers the difference between the light absorbance through the liquid prepolymer resin and the solid cured polymer. This model is used to explain why a part manufactured with 10 µm layer thickness has double or more the strength, the storage modulus, or the degree of monomer conversion of a part of 50 µm layer thickness for the same exposure time and LED power. 

Original terminology, the Irradiance affected zone (IAZ), is introduced, which defines the number of layers affected by the projected irradiance for particular exposure time and is a function of the process parameters and the material working curve constants. The IAZ sets a limit on the number of layers considered in calculating the accumulated energy for each of the previously cured layers and also defines the minimum feasible size of horizontal channels to be manufactured for a specific material at process parameters. A horizontal curing model is discussed and used to assess the minimum feasible size for different geometrical results and also to show that, as the exposure time increases, the diameters of the channels decrease, and the diameters of solid bars increase.

We present an innovative experimental methodology to evaluate the constants of the working curve for the multi-layer curing model. The results show that LED power affects the cured depth for the same exposure time. Also, the light penetration through a liquid prepolymer was found to be higher than through a cured polymer, which explains the necessity to consider these differences in the accumulated energy model. The machine irradiance was characterized, and it was found that the PµSLA have significant irradiance irregularities. To minimize the effect of the irradiance irregularities on the measured responses, a distinct region on the build area with tolerable irradiance difference was used strictly to manufacture all the test specimens. For the material properties, the layer thickness was found to be the most significant parameter controlling the process outcomes. The next most significant parameter was exposure time. LED power was the least significant process parameter. It is crucial to select the proper process parameter to achieve the geometrical dimensions required while having enough green strength for the part to hold itself against its own weight, separation force, and post-processing. A generic empirical logarithmic regression is proposed to predict the different material properties based on the process parameters and the material working curve constants, represented by the accumulated energy. The proposed model facilitates the development of prediction models based on simple experimental procedures. Because this model is analogous to the working curve equation, it can define the critical amount of energy required to start developing the different material properties.

A geometry artifact was designed to study the effect of the process parameters on various features of different sizes. It was found that horizontal channels smaller than the irradiance affected zone will not be feasible physically to be manufactured. The layer thickness and exposure time are the most significant parameters for both the horizontal channels and overhangs, while the exposure time is the most significant for vertical channels and bars. The LED power was the least significant parameter for all the features. The results of this paper can be used for the general optimization of the process in terms of geometry, speeding up the process by decreasing the exposure time without harming strength. It also can be used for estimating an initial solution for dedicated geometry optimization algorithms.

## Figures and Tables

**Table 1:** Values of the process parameters for three designed experiments.

Layer Thickness								
50 µm			25 µm			10 µm		
#	Time (s)	Power	#	Time (s)	Power	#	Time (s)	Power
1	2	HI	1	1.8	HI	1	1.6	HI
2	2	LO	2	1.8	LO	2	1.6	LO
3	1.6	HI	3	1.4	HI	3	1.3	HI
4	1.6	LO	4	1.4	LO	4	1.3	LO

**Table 2:** Measured irradiance values across the build area (mW/cm^2^).

Position	Left	Center	Right
Top	12.5	17	12.5
Middle	14.3	18.7	14.7
Bottom	15.6	19	16.2

**Table 3:** Constants values for material property regression model.

Material Property (γ)	Constant	HI Power	LO Power
UTS (MPa)	$C_{1}$ (MPa)	12.86	12.7
	$C_{2}$ (mJ/cm^2^)	23.27	31
Storage Modulus (MPa)	$C_{1}$ (MPa)	240	294
	$C_{2}$ (mJ/cm^2^)	0.806	2
DOC (AU)	$C_{1}$ (AU)	0.281	0.176
	$C_{2}$ (mJ/cm^2^)	8.7	3.2

**Table 4:** Average measured horizontal (H) and vertical (V) diameters of the horizontal channels and width of the vertical bars.

*d_z_*	#	Horizontal Circular Channels (µm)								Vertical Square Bars (µm)					
		750 H	750 V	650 H	650 V	500 H	500 V	350 H	350 V	1000	750	500	250	200	150
50 µm	1	705	614	636	564	471	468	343	323	1022	769	509	261	200	**×**
	2	752	669	640	623	501	468	339	333	1034	776	503	240	175	**×**
	3	733	683	634	637	505	488	349	356	1017	773	505	257	199	138
	4	762	666	683	656	544	500	390	**×**	1002	748	474	229	**×**	**×**
25 µm	1	732	624	575	573	**×**	**×**	**×**	**×**	1055	796	519	263	220	**×**
	2	766	660	643	660	541	495	**×**	**×**	996	751	428	209	**×**	**×**
	3	665	665	602	588	**×**	**×**	**×**	**×**	1027	767	506	240	202	**×**
	4	667	671	613	584	472	443	**×**	**×**	1057	791	517	258	207	**×**
10 µm	1	747	626	681	586	**×**	**×**	**×**	**×**	979	726	409	**×**	**×**	**×**
	2	782	666	758	587	**×**	**×**	**×**	**×**	929	710	374	**×**	**×**	**×**
	3	738	646	663	584	**×**	**×**	**×**	**×**	993	749	450	**×**	**×**	**×**
	4	805	675	707	575	**×**	**×**	**×**	**×**	980	730	445	**×**	**×**	**×**

**Table 5:** Average measured width of the vertical square channels and the thickness of the overhangs.

*d_z_*	#	Vertical Square Channels (µm)						Over Hangs (µm)						
		750	650	500	350	200	150	1000	750	500	250	200	150	100
50 µm	1	775	666	511	362	201	127	1050	790	570	270	234	176	105
	2	756	673	501	353	179	**×**	1038	787	554	263	226	129	83
	3	784	676	511	345	200	142	1011	778	543	256	220	133	**×**
	4	776	677	519	370	199	168	1002	775	533	249	208	132	60
25 µm	1	778	657	501	296	**×**	**×**	1024	811	562	301	235	177	111
	2	813	701	534	387	223	167	1091	787	544	268	246	177	90
	3	777	689	527	361	196	125	1053	809	526	261	210	181	122
	4	775	662	492	339	178	**×**	1074	821	557	260	200	120	**×**
10 µm	1	866	738	577	406	**×**	**×**	1074	817	600	293	229	145	**×**
	2	864	768	624	441	**×**	**×**	1060	805	583	286	216	**×**	**×**
	3	833	706	555	357	**×**	**×**	1055	796	584	279	230	**×**	**×**
	4	853	701	570	390	229	**×**	1081	822	525	264	177	**×**	**×**
